# Supplementary material for: Prognostic Role of Soluble and Extracellular Vesicle-Associated PD-L1, B7-H3 and B7-H4 in Non-Small Cell Lung Cancer Patients Treated with Immune Checkpoint Inhibitors
Source: Cells. 2023 Mar 8;12(6):832. doi: 10.3390/cells12060832 (PMC10047289; doi:10.3390/cells12060832)
Supplement: Supplementary file 1 [file cells-12-00832-s001.zip › cells-2213101-supplementary.pdf]

Supplementary Materials: Prognostic role of soluble and extracellular vesicle-associated PD-L1, B7-H3 and B7-H4 in non-small cell lung cancer patients treated with immune checkpoint inhibitors

Carlo Genova[1,2+], Roberta Tasso[3,4+], Alessandra Rosa[5+], Giovanni Rossi[6,7], Daniele Reverberi[8], Vincenzo Fontana[5], Silvia Marconi[9], Michela Croce[10], Maria Giovanna Dal Bello[11], Chiara Dellepiane[6], Marco Tagliamento[2], Maria Chiara Ciferri[3], Lodovica Zullo[6], Alessandro Fedeli[12], Angela Alama[9], Katia Cortese[3], Chiara Gentili[3,4], Eugenia Cella[6], Giorgia Anselmi[13], Marco Mora[13], Giulia Barletta[6], Erika Rijavec[14], Francesco Grossi[14], Paolo Pronzato[6], and Simona Coco[9]

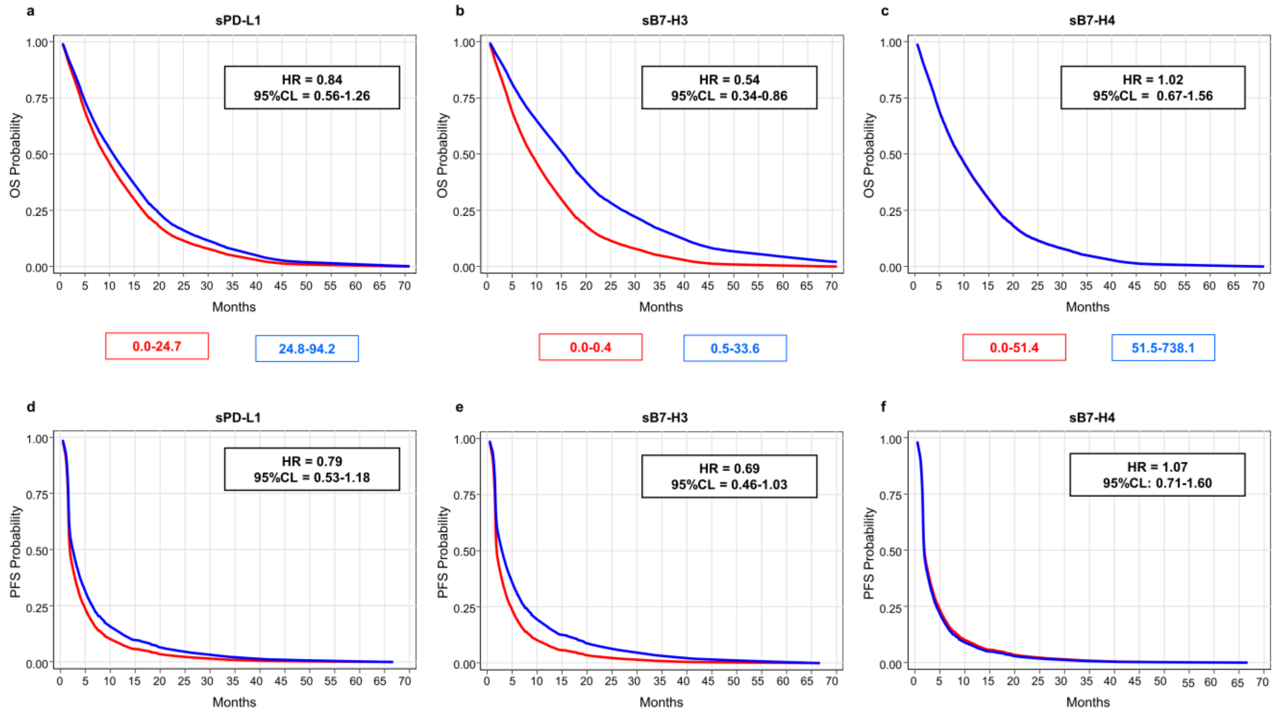

Figure S1. Overall (**a, b, c**) and progression-free (**d, e, f**) survival probabilities estimated through the multivariable Cox regression analysis in 126 patients of Nivo cohort stratified according to biomarker median values: PD-L1 = 24.2 pg/mL (**a, d**); B7-H3 = 0.5 ng/mL (**b,e**); B7-H4 = 63.9 pg/mL (**c,f**). Legend - HR: hazard rate ratio adjusted for gender, age, cycles of therapy, ECOG-PS and histotype; 95%CL: 95% confidence limits for HR.

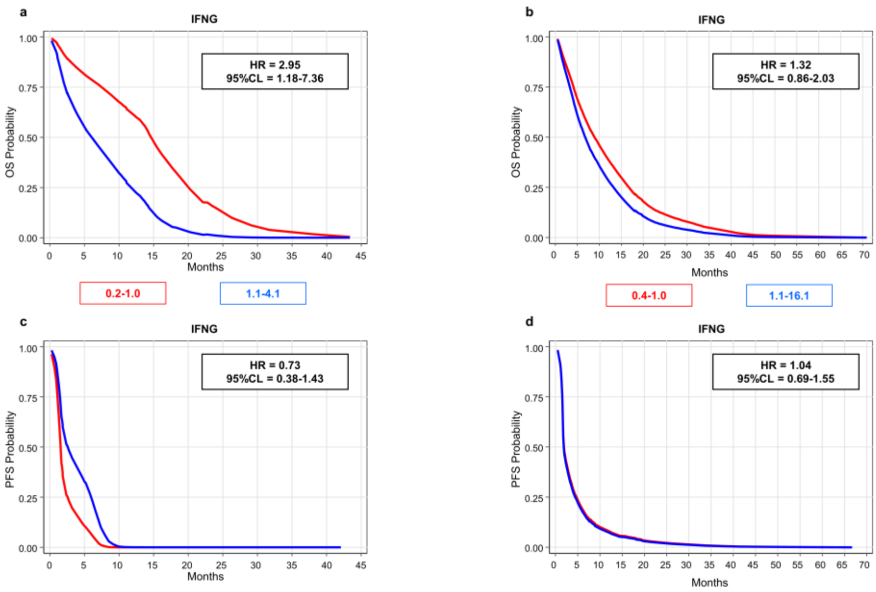

Figure S2. Overall (**a, b**) and progression-free (**c, d**) survival probabilities estimated through the multivariable Cox regression analysis in 56 patients of Pembro cohorts (**a, c**) and 126 patients of Nivo cohort (**b, d**) stratified according to IFNG median value (1.0 pg/mL). Legend - HR: hazard rate ratio adjusted for gender, age, cycles of therapy, ECOG-PS and histotype; 95%CL: 95% confidence limits for HR.

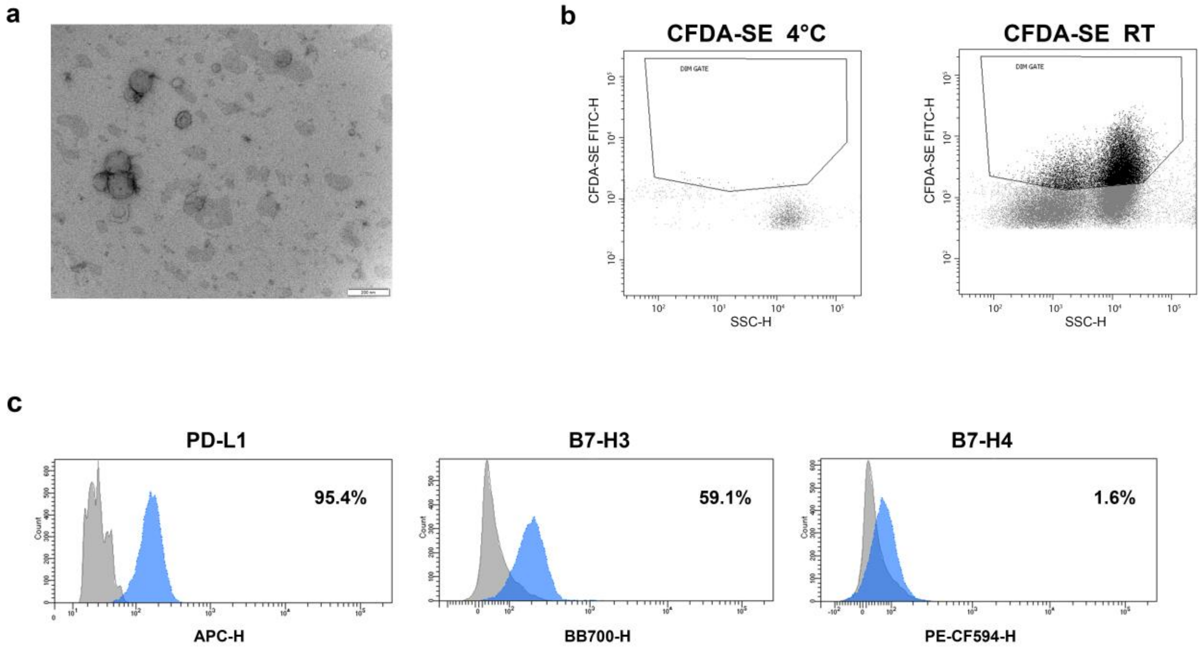

Figure S3. (**a**) Representative TEM image of plasma EVs; scale bar: 200 nm. (**b**) Flowcytometry strategy used to characterize plasma EVs. Black areas identify CFDA-SE positive events. EVs were stained with CFDA-SE at 4°C as "blank tube" (left panel) to define the appropriate dimensional gate when considering EVs stained with CFDA-SE at room temperature (right panel). (**c**) Representative flow cytometry analysis of EVs isolated from the plasma of a patient from the Pembro cohort. Areas under the blue lines identify EVs positive for PD-L1, B7-H3 and B7-H4. Areas under the grey lines indicate the interactions of EVs with corresponding non-reactive immunoglobulin of the same isotype

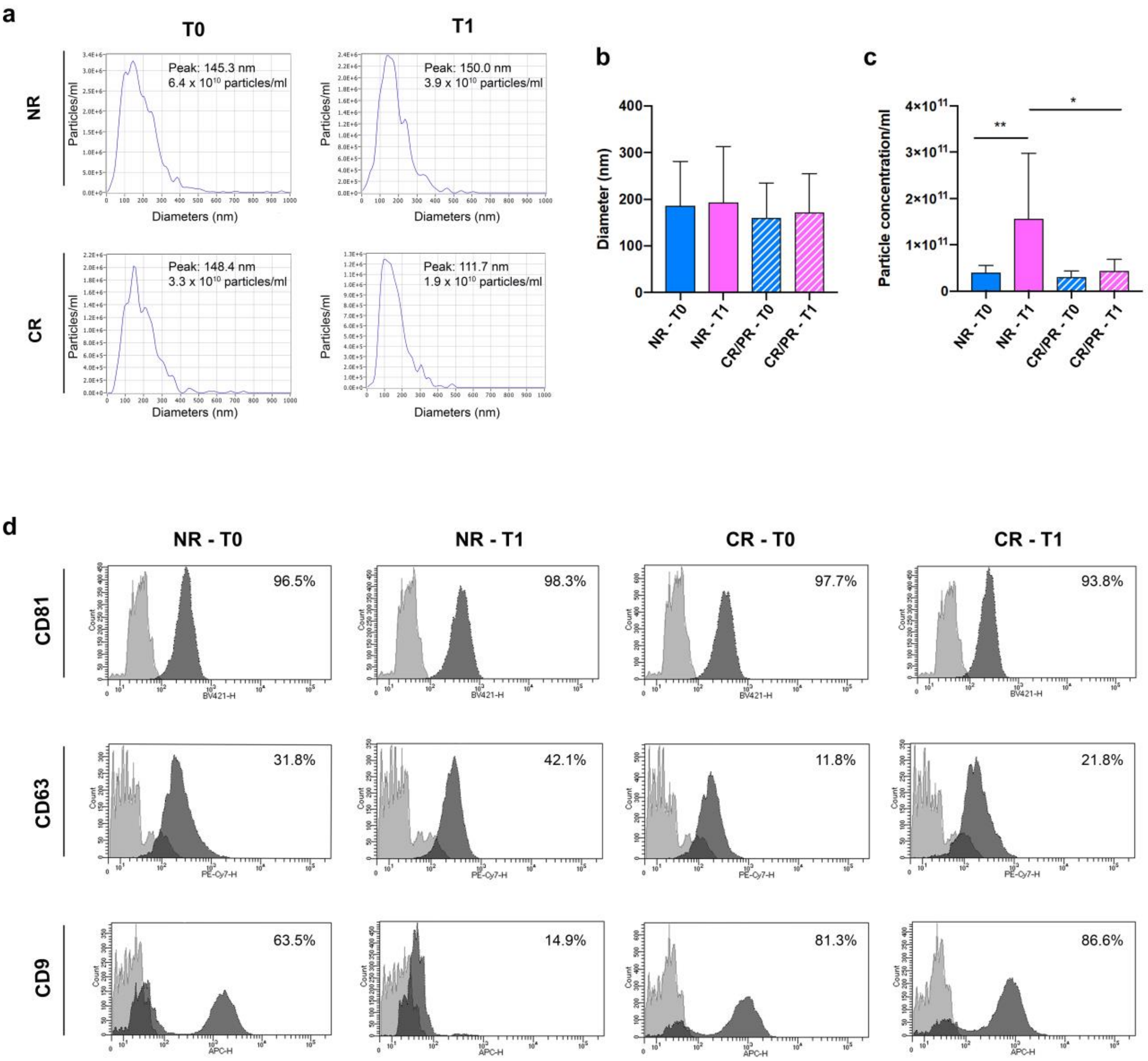

Figure S4. (**a**) Representative histograms showing the size distribution and the particle concentration by NTA of EVs isolated from both a NR and from a patient underwent a complete response. The analysis has been performed evaluating the EVs prior to therapy (T0) and at CT assessment of first response (T1) after therapy. (**b, c**) Histograms representing the diameter (nm) (**b**) and the concentration/ml (**c**) of EVs derived from 11 NR and 9 patients underwent a complete or partial response (CR/PR), at both T0 and T1. (**d**) Flow cytometry analysis of EVs isolated from NR and CR patients, at both T0 and T1. Areas under the black lines identify vesicles reacting with CD81, CD63, and CD9. Areas under the grey lines indicate the interactions of EVs with corresponding nonreactive immunoglobulin of the same isotype

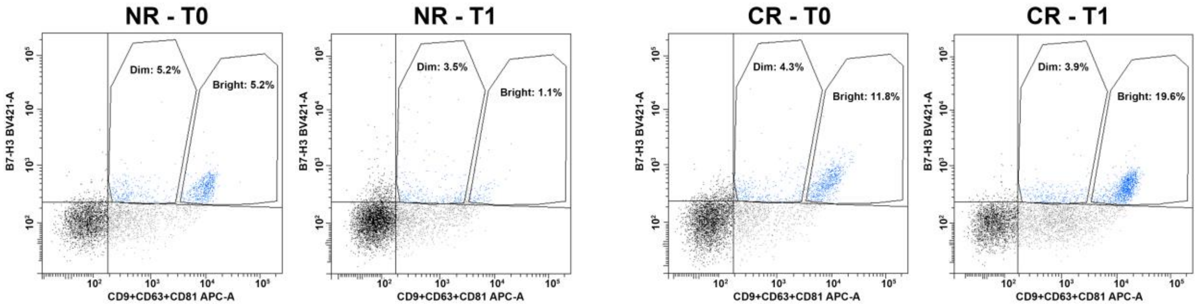

Figure S5. Representative dot plots of NR and CR patients indicating B7-H3 positive EVs falling in the Dim and Bright dimensional gates.
